# Neurobiology of Aggression—Review of Recent Findings and Relationship with Alcohol and Trauma

**DOI:** 10.3390/biology12030469

**Published:** 2023-03-20

**Authors:** Michael Fritz, Sarah-Maria Soravia, Manuela Dudeck, Layal Malli, Marc Fakhoury

**Affiliations:** 1 School of Health and Social Sciences, AKAD University of Applied Sciences, 70191 Stuttgart, Germany; 2 Department of Forensic Psychiatry and Psychotherapy, Ulm University, BKH Günzburg, Lindenallee 2, 89312 Günzburg, Germany; 3 Department of Natural Sciences, School of Arts and Sciences, Lebanese American University, Beirut P.O. Box 13-5053, Lebanon

**Keywords:** alcohol, aggression, trauma, neurobiology, imaging, genes, neurotransmitters, immune markers

## Abstract

**Simple Summary:**

Aggression can be viewed as any form of behavior, such as physical or verbal, that is intended to cause harm or injury to another living being. This form of behavior has seen its prevalence increase over the past few decades, affecting individuals of all ages and accounting for more than 1.3 million deaths worldwide. As such, the scientific community invested a lot of effort into better understanding its risk factors and mechanisms. Over the past few years, several studies have been conducted in animals and humans for this purpose, with the overall consensus that aggression can be precipitated by several risk factors, including biological and environmental. There is also evidence showing that aggression is linked with alcohol consumption and trauma exposure. However, despite significant progress in research, the mechanisms through which these factors lead to aggressive behaviors are poorly understood. This review provides the current state of knowledge regarding the neurobiology of aggression and highlights recent evidence discussing its relationship with alcohol and trauma.

**Abstract:**

Aggression can be conceptualized as any behavior, physical or verbal, that involves attacking another person or animal with the intent of causing harm, pain or injury. Because of its high prevalence worldwide, aggression has remained a central clinical and public safety issue. Aggression can be caused by several risk factors, including biological and psychological, such as genetics and mental health disorders, and socioeconomic such as education, employment, financial status, and neighborhood. Research over the past few decades has also proposed a link between alcohol consumption and aggressive behaviors. Alcohol consumption can escalate aggressive behavior in humans, often leading to domestic violence or serious crimes. Converging lines of evidence have also shown that trauma and posttraumatic stress disorder (PTSD) could have a tremendous impact on behavior associated with both alcohol use problems and violence. However, although the link between trauma, alcohol, and aggression is well documented, the underlying neurobiological mechanisms and their impact on behavior have not been properly discussed. This article provides an overview of recent advances in understanding the translational neurobiological basis of aggression and its intricate links to alcoholism and trauma, focusing on behavior. It does so by shedding light from several perspectives, including in vivo imaging, genes, receptors, and neurotransmitters and their influence on human and animal behavior.

## 1. Introduction

Aggression is a universal trait; it exists in all animals, including human beings and primates. In its essence, aggression functions to harm others [1]; its motives, however, are more facetted. Aggression occurs to protect and secure resources like food, group members, packs, families, tribes, and territory [2], factors that ultimately allow growth and survival. Its “success story” and its evolutionary psychological value appear indisputable. In fact, systematic aggression in the form of war was invented around 3250 years ago in Tollense Valley, Germany [3], and Megiddo, Israel [4], respectively, and is still present even in the world′s most developed countries today.

Aggression can be reactive, instrumental (proactive), or appetitive in its nature. Reactive aggression subsumes verbal and physical violence in reaction to threat, attack, or provocation [5]. Instrumental aggression includes behavior that utilizes violence to reach certain aims [6], whereas appetitive aggression means violence for the sake of violence and pleasure [7]. From a dimensional perspective, aggression can be adaptive or maladaptive, meaning it can be rightful from an ethical perspective or socially condemnable and out of context [8]. If threatened with grave unprovoked physical harm, no one will dispute physical aggression to defend oneself. Contrarily, intermittent explosive disorder, extortion to gain riches, or sadistic murder are deemed wrong.

Intriguingly, these different means of aggression are not limited to human beings. Our evolutionary neighbors, monkeys and human primates, exemplify such different strategies as well. Here, violence regulates hierarchies within gorillas and chimpanzees and represents means to interact with other groups [9]. From a gender perspective, there do exist sex differences in human aggression. However, they are not as striking as one may think; Been and colleagues [10] call it even an “archaic belief”, women would not readily engage in social aggression. In fact, men and women score equally regarding verbal aggression; however, men tend to be more physically aggressive, whereas women express violence more indirectly [11].

To make matters worse, confounding variables appear to facilitate the probability of aggressive behavior. Among those, the most prominent factor is presumed to be alcohol [12]. The trait “anxiety” is also thought of as another modulator. In line with this, the work of Florek et al. [13] demonstrated an interaction between anxiety, alcohol intake, and aggression during the present SARS-CoV-II pandemic. Nevertheless, animal experiments tainted this point of view. Fearful rats (high anxiety-related behavior; HAB) display generally less aggression than fearless rats (low anxiety-related behavior; LAB, [14]). A factor bridging this observational difference on a human level may be trauma or traumatic stress. An interaction between trauma and heightened aggression was shown for both men [15] and women [16] and previously linked to a functional genetic polymorphism [17]; a possible neuroanatomical correlate for such an interaction was recently deciphered in rodents [18].

**Figure 1:**
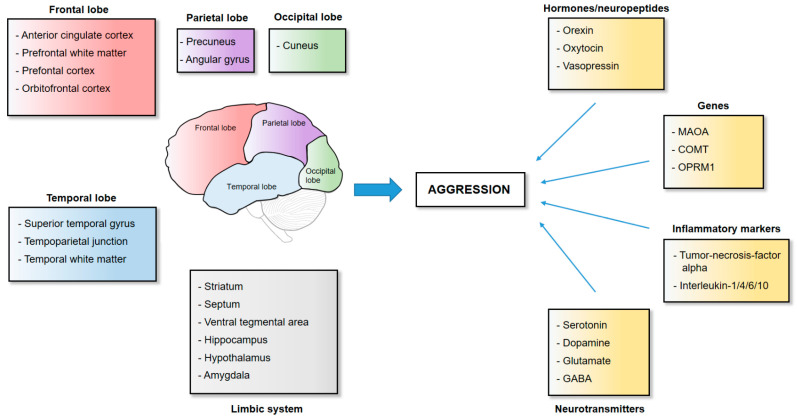
Neuroanatomical and genetic basis of aggression in humans and animals. Aggressive behavior is mediated by several brain regions originating from the frontal, parietal, occipital, or temporal lobe and from regions part of the limbic system. Other factors that influence human and animal behavior include genes, inflammatory markers, hormones, neuropeptides, and neurotransmitters.

If this introduction may draw one conclusion, then that of a very complex one which can be looked upon from various angles. In this light, our review attempts to summarize recent findings of this vivid ménage à trois by starting out with the anatomical, neurotransmitter, and genetic basis of aggression (summarized in Figure 1) and moving on to alcohol and trauma as modulating factors. Methodologically we used PubMed^®^ and Google Scholar, narrowing the time window in the first approach down to 2012–2022, gradually expanding the timeframe if no or very few recent publications could be identified. Fundamental theoretical work was searched specifically. The Boolean operator “AND” was applied to further specify searches. All authors did their best to collect the most recent information regarding this topic, focusing mostly but not exclusively on the last decade but are wholly aware of the possibility of having failed this task.

## 2. The Neuroanatomy of Aggression

### 2.1. Frontal Cortical Structures

Where to start? Principally, where most neuroscientific research concerning behavior and decision-making starts—the cortex. Cortical structures are fundamentally associated with aggression; amongst those, structural and functional deviations in the prefrontal and medial temporal regions have been extensively investigated and meta-analyzed [19,20]. For instance, the ability to control aggression was linked to the anterior cingulate cortex (ACC) in both rodents and human beings. Volumetric and neuronal activity alterations were found to correlate with social misconduct and callousness in adolescents [21,22] as well as with aggression in mice [23]. Additionally, van Heukelum and colleagues [23] aimed to provide a link between ACC activation and the suppression of pathological aggression. The study applied a chemogenetic approach to activate the ACC and consequently repressed pathological aggression almost completely in mice.

Furthermore, sub-cortically both prefrontal and temporal abnormal white matter integrity was reported in 15 male convicted rapists compared to controls [24]. In light of these findings, interventions targeting prefrontal areas seemed to be expedient. To date, four studies applied transcranial direct current stimulation (tDCS) to prefrontal areas attempting to modulate aggression and aiding a causality understanding. However, the results were mixed. Stimulating the right dorsolateral prefrontal cortex (dlPFC) reduced proactive aggression [25], whereas stimulating the left dlPFC triggered the opposite effect if participants were already in a negative affective state [26]. On the other hand, stimulating the inferior prefrontal cortex yielded no result at all [27]. Still, bilaterally stimulating the dlPFC caused participants to view aggression and sexual violence as more morally despicable and decreased the intentions of committing aggression [28]. Conversely, lesions to the dlPFC promoted physical aggressiveness, as demonstrated in a study with Vietnam War veterans who received penetrating brain injuries [29].

A different mean of stimulation, continuous Theta-burst transcranial magnetic stimulation (cTBS), further cooperated an involvement of dlPFC activity in aggression. Interestingly, inhibiting the left dlPFC caused healthy volunteers to act reactively and proactively more aggressively, contradicting the findings described above [30]. An activating cTBS protocol across both hemispherical dlPFCs, however, led to an increased accuracy in of identifying emotions and reduced likelihood of aggression in participants with an antisocial personality disorder [31].

The work of Chester and colleagues [32,33,34] implicated two additional parts of the prefrontal cortex (PFC) in the complex matter of aggression; the ventromedial (vm) and the ventrolateral (vl) PFC. The vmPFC showed volumetric and gray matter deficits in the context of heightened aggression, whereas augmented activation of the vlPFC during rejection could be linked to increased retaliating aggression. Additionally, an overall decreased gyrification of the frontal lobe, as well as prefrontal thinning, were related to intensified aggressive behavior in school children [35]. The involvement of these PFC areas in circuits of escalated aggression has been highlighted in rodents as well [36,37].

Finally, another subregion of the prefrontal cortex, the orbitofrontal cortex (OFC), seems to play a role as well, especially given the famous case of Phineas Gage [38]. It does not surprise that across species, OFC abnormalities or chemogenetic inactivation are associated with increased aggression [39,40,41].

### 2.2. Temporal Cortical Structures

An initial report in a small population of highly violent criminals was the first to implicate metabolic abnormalities of lower medial temporal lobe structures in aggression [42]. Volumetric differences in adolescents with a conduct disorder diagnosis were then described by Kruesi and colleagues [43]. Next, a review of 17 imaging studies further strengthened the involvement of temporal structures in aggressive behaviors [44], with the recent work of Buadas-Rotger and colleagues [45] linking angry face reactivity in the superior temporal gyrus (STG) to task-related aggression. On a network level, this hyperreactivity was related to activation in the amygdala. Hence it is of no surprise that the neuro-moral theory of antisocial behavior emphasizes the involvement of the superior temporal gyrus, the angular gyrus, and the tempoparietal junction as key brain areas [46]. A recent meta-analysis of 2022 further confirmed this role of temporal lobe structures activities in aggression-prone individuals [47].

### 2.3. The Striatum

The striatum is a forebrain structure divided into a ventral and dorsal part, which is in humans further separated into the caudate and the putamen. Whereas the first receives strong dopaminergic inputs from the ventral tegmental area (VTA), the latter one is mostly innervated by the substantia nigra (dorsal part). The ventral part of the striatum is thought to facilitate learning, motivated behavior, and reward evaluation [48]. Unsurprisingly, imaging studies revealed functional abnormalities during increased retaliation-oriented aggression [33]. Additionally, violent offenders responded to provocations with high striatal reactivity [49]. Finally, increased volumes of the caudate, the putamen, and the nucleus accumbens (NAc; ventral striatum) were associated with augmented reactive aggression [50], while increased dorso-striatal activity measured with functional magnetic resonance imaging (fMRI) revealed a higher motivation to punish unfairness [51]. Interestingly, recently developed animal models demonstrated a “highly rewarding” aspect of aggression and violence to be mediated in dopaminoceptive D1-neurons in the NAc [52]. This “winner effect” describes the phenomenon that victorious mice are more likely to maintain successful aggressive behaviors [53], prefer the compartment associated with the victory [54], and respond in an operant conditioning paradigm with lever-pressing to encounter an intruder mouse to fight it [55].

### 2.4. The Limbic System

Hostility and anger combined in one outburst is called rage [56]; such rage is known to originate in the septum across species [57,58,59], a limbic structure in the epicenter of a vast neuronal aggression network connecting the amygdala, the VTA, and the hippocampus, and even extending to the PFC [60,61]. Modern neuroscientific circuitry studies based on optogenetics in mice revealed glutamatergic inputs from the CA2 region of the hippocampus and dopaminergic innervations from VTA to the septum to promote aggression [62,63]. On the other hand, overall inhibition and activation of the descending outputs from the lateral part of the septum (LS) to the hypothalamus were shown to modulate aggression bidirectionally. Inhibition of LS increased aggression through a GABAergic mechanism in rodents [64], whereas activation of the LS-to-hypothalamus connectivity reduced aggressive behavior [59], indicating an intricate sub-circuitry within the septum and its subdivisions.

Longstanding experimental evidence implicated hypothalamic structures in aggression and violence independent of species (for review, see [65]), even coining the term “hypothalamic attack area”, located at the ventrolateral pole of the ventromedial hypothalamic nucleus (VMHvl; [66]). Single-unit electrophysiology and population optical recording demonstrated an increase in activity in the VMHvl immediately before the initiation of an attack [55]. Silencing this brain area abolished inter male aggression while activating it led to off-target attacks on inanimate objects and female mice [67]. Additionally, optogenetic stimulation of the prefrontal-cortical innervations to the more mediobasal and lateral parts of the hypothalamus was also found to be crucial for initiating violent bites in intermale aggression in mice [68].

In human beings, the “hypothalamic attack area” is suggested to be located in the more posteromedial part of the hypothalamus, also called the “Triangle of Sano” [69]. However, even though initial surgical interventions suggested promising results [70], deep brain stimulation of the posteromedial hypothalamus during pathological aggressiveness was of limited success [71,72,73,74].

Another brain area implicated in aggressiveness—and where our memories are formed—is the hippocampus. A recent chemogenetic approach, for instance, demonstrated ventral hippocampal control of stress-induced aggression in mice [75]. From a human perspective, a study with 67 participants suffering from intermittent explosive disorder (IED) revealed morphometric deformation in the hippocampus, which according to the authors, represented significant neuronal loss [76]. Along this line of thinking, the volumetric ratio between the hippocampus and amygdala has been implied in many psychopathologies, including heightened aggression [77]. On a functional level, Nikolic and colleagues [47] reported increased activity in both the left hippocampus and amygdala in individuals with a known history of violence during anger-eliciting tasks. Increased connectivity between the right hippocampus and the cingulate cortex could be associated with aggressive behavior of patients with a history of mild concussions [78].

The amygdala is a complex brain area that can be divided into a central and medial part on a microanatomical level. It plays a role in a multitude of cognitive functions like learning and memory. However, the amygdala is also implicated in several pathological conditions like anxiety disorder or addiction. Haller suggested in his review [79] that predatory aggression is especially mediated through the central part of the amygdala. In line with this thought, chemogenetic experiments identified a neuronal subtype in the posterodorsal-medial part of the amygdala controlling aggressive behavior in both male and female mice [80], while estrogen receptor-carrying neurons in the posterior amygdala signaling to the hypothalamus fulfilled the same role in inter-male aggression [81]. An optogenetic approach identified the posterior ventral segment of the medial part of the amygdala to be essential in primed aggressive behavior [18]. Here optogenetic high-frequency stimulation mimicked aggressive encounters and subsequently increased aggressive behavior in mice. Overall chemogenetic inhibition of the medial amygdala abolished lactating female-to-male mice aggression [82]. In human beings, amygdala reactivity to fearful facial expressions was linked to impulsive aggression [83], while children showing pronounced aggressive behavior had a decreased amygdala volume compared to controls [35]. In line with this observation, another study reported significant structural deformations of the amygdala in IED patients [76]. A longitudinal study with 503 participants running from first grade to the age of 26 provided further evidence of a link between decreased amygdala volume, violence, and even psychopathic traits [84]. Nonetheless, a recent study failed to link amygdala volumes to scores in the revised Psychopathy Check List (PCL-R; [85]). In contrast, a longitudinal observational study from Saxbe et al. [86] linked aggression and externalization running through family lines to a volumetric increase in the right amygdala. Fundamental work from Raine and colleagues [87] went even further and differentiated brain metabolic activity regarding proactive and impulsive aggression in convicted murderers. PET scans revealed that both groups had heightened metabolism in the right limbic system (i.e., amygdala, hippocampus, and midbrain), although being “excessively” augmented in predatory murderers. Accordingly, the work of da Cunha-Bang [49] reported increased amygdala reactivity to provocations in violent offenders. In another set of studies, Heesink et al. [88], Varkevisser et al. [89], and Ibrahim et al. [90] pointed out that the connectivity between the amygdala and PFC was decreased in war veterans as well as children with aggression issues. Conversely, Saxbe et al. [91] found such a decrease in connectivity to be restricted to the amygdala and the posterior cingulate cortex. On the other hand, resting-state fMRI revealed an overall increase in amygdala connectivity but pronounced severity in aggression was associated with a global reduction in connectivity in the vmPFC and dorsal ACC [92]. However, the trait “aggressiveness” did not relate to amygdala-OFC connectivity [93].

Finally, the VTA in itself and especially its primary dopaminergic cell population projecting to the LS were recently demonstrated to powerfully modulate aggression in rodents [94]. On a molecular level, dopamine 2 (D2)-receptors expressed on GABAergic neurons were identified as key players mediating such behavior [63]. Interestingly, winning “mice territorial disputes” was shown to prime VTA-to-NAc innervations, making the occurrence of aggression more likely [95]. Along with these types of observations, a recent study was able to link VTA hyper-reactivity to impulsive aggression and anti-sociality [96]. It is also worthwhile to note that Been and colleagues [10] underlined the VTA as a key node in the female rather than male circuitry of aggression.

### 2.5. The Parietal and Occipital Lobe

The precuneus, as part of the parietal lobe, is a brain area implied in a variety of complex functions and is part of the default mode network. Unsurprisingly, a recent meta-analysis of fMRI studies confirmed a significantly changed connectivity of the precuneus and frontal brain structures in aggression-prone individuals [47], as well as in juvenile violent offenders [97] and adolescents displaying disruptive behavior [98]. Additionally, increased neuronal reactivity to socio-emotional stimuli in the precuneus was linked to aggression [99,100].

The cuneus, a part of the occipital lobe, is a central part in the evaluation of threat. In line with this, work from Varkevisser and colleagues [89] and Heesink and colleagues [88] demonstrated increased connectivity between the ACC and the cuneus and its overall activation in impulsive-aggressive combat veterans. Interestingly, the recent work of Zhu and colleagues [101] reported a link between the cortical thickness of both the parietal and occipital lobes and overall aggressiveness in 240 participants. Finally, Nikolic and others [47] reported an overall reduced activity of the left occipital cortex in their recent meta-analysis of imaging studies in aggressive individuals.

## 3. The Neurotransmission and Genetics of Aggression

### 3.1. Serotonin, Dopamine and Their Degradation

Serotonin (5-hydroxytryptamine; 5-HT) plays an important role in higher cognitive functions as well as mood and is considered “the” most important neurotransmitter contributing to aggression [102]. It can act through 15 overall receptors divided into 7 families: 5-HT_1_ to 5-HT_7_. Of those, are all but 5-HT_3,_ which is a ligand-gated sodium channel, are either excitatory or inhibitory G protein-coupled receptors [103].

The so-called “Serotonin deficiency” hypothesis dated to work from Linnoila and others [104], where 5-hydroxyindoleacetic acid (5-HIAA), a serotonin metabolite, turned out to be significantly lower in the cerebrospinal fluid of violent offenders. Yet, despite that this inverse relation was repeatedly described as “strong” or “well established”, a recent meta-analysis of studies including more than 6500 participants demonstrated not more than a small, marginally significant effect [105].

Despite such a modest effect, modern neuroscientific tools keep pointing towards a serotonergic role in aggression. Since the discovery that the central nervous system has its own enzyme to synthesize serotonin (Tryptophan hydroxylase-2 (Tph-2); [106]), it was shown that a partial or complete knockdown led to more aggressive behavior in rodents [107,108,109,110] and could be linked to a diminished 5-HT_1A_ receptor sensitivity [111]. On the other hand, highly aggressive wild-derived mice had higher mRNA levels of Tph-2 than other laboratory animals [112]. In human beings, functional polymorphisms like TPH2 G-703/T (G/G instead of G/T and TT) could be linked to anger-related traits in Korean women [113] and an anger-trait and negative OFC volume interaction [114], as well as changed in OFC serotonin synthesis in aggressive subjects [115]. Similarly, Laas and colleagues [116] found in Estonian adolescents the T/T variant carriers of the TPH2 G-703/T polymorphism show the least aggressiveness.

In line with Tph-2 knockdown results, the pharmacological depletion of serotonin itself also increased the attack duration in mice [117]. On a receptor level, altering serotonergic cell signaling either by genetic overexpression of the serotonin 1a receptor (5-HT1A receptor) in adult mice or pharmacological 5-HT1A receptor activation led to an immediate increase in aggressive behavior [118]. Conversely, systemic administration of the 5-HT1A receptor antagonist 8-OH-DPAT inhibited social-isolation-enhanced aggression in mice [119]. Specific 5-HT1A receptor activation in the hypothalamus, on the contrary, had sex-dependent behavioral effects in hamsters. While female hamsters reacted aggressively, male hamsters reduced their aggressiveness [120]. Further complicating the matter, microinjections of the novel 5-HT1A receptor agonist into the OFC had anti-aggressive properties, thereby opposing most of the findings described above [121]. Finally, a recent pharmacological study implicated the 5-HT2C receptor in aggressive behavior as well; 5-HT2C receptor antagonism with the compound SB243213 reduced social-isolation-induced aggression [122].

In humans, multiple single nucleotide polymorphisms (SNPs) in the 5-HT2A receptor have been related to antisocial and aggressive behavior in young adults diagnosed with conduct disorder and in two traditional and one industrial Russian populations [123,124]. Furthermore, post-mortem investigations of the OFC in nine individuals with a history of antisocial behavior and substance use disorder (SUD) revealed increased 5-HT2A receptor levels compared to a SUD-only group and healthy controls [125]. However, frontal cortex 5-HT2A receptor binding density could not be linked to trait aggressiveness or impulsivity in healthy human subjects [126]. Interestingly, the same research group did report a positive correlation between striatal 5-HT1B receptor binding and the trait anger in violent offenders [127]. Somewhat surprising, given the finding of da Cunha-Bang and colleagues [126], the 5-HT1B receptor SNP rs6296 could only be related to childhood aggressiveness but did not associate with aggression during adulthood in a Finnish population [128]. In a third study, da Cunha-Bang and colleagues [129] used the fact that 5-HT4 receptor binding could be used as a proxy for 5-HT levels. Here, total 5-HT4 receptor radiotracer binding correlated highly with self-reported aggression levels in men. In other words, the lower the global serotonin level, the higher the self-reported aggression score.

Dopamine is a neurotransmitter attributing salience to stimuli, generating perceptional valence, and thereby promoting motivation to act. It also helps animals and human beings to obtain rewards and avoid negative outcomes. Dopamine asserts control over neuronal activity via two groups of dopaminergic receptors—the d1-like and the d2—like family. The first group consists of d1- and d5-receptors, the latter one of d2-, d3-, and d4-receptors [130].

Mahadevia and colleagues [63] were able to pinpoint augmented aggression in mice to a d2-receptor-mediated mechanism in the LS, while aggression self-administration and aggressive behavior were controlled by NAc d1-receptor neurons [52] through the transcription factor ΔfosB [131]. On the other hand, d1 receptors were found to be significantly reduced in the frontal cortex [132], with lower dopamine concentrations in the PFC as well as the hippocampus in highly aggressive rodents [133]. These findings were supported by a large gene expression investigation revealing Slc-gene-related alterations linked to repeated victorious aggressive encounters in mice [134]. In this study, Slc genes linked amongst others to the dopamine transporter were significantly downregulated in the VTA as well as the NAc, with upregulation patterns in the PFC. Somewhat consistently, methylphenidate, a dopamine transporter blocker, reduced aggression and promoted sociability in mice [135]. Bridging these differences, the administration of a novel dopamine stabilizing compound (-)-OSU61612 had reportedly anti-aggression properties in socially isolated rats [136].

The results outlined above appeared to be translatable since the work of Pape et al. [137] concluded that methylphenidate led to a normalization of NAc connectivity to cortical regions linked to moral decision-making and attention in adolescents with disruptive behavior disorder. However, behavioral changes were not assessed during the exploration.

An array of investigations looking at variations in dopamine genes revealed a significant correlation with aggressive behaviors. In children, for instance, the seven-repeat allele variant of the d4 receptor gene served as a significant predictor for aggression and violence in a four-year follow-up study [138]. Similar findings were described in young adults by Buchmann et al. [139] and Wu and Barnes [140], who were able to link the variant to both aggression and altered cortisol responses, as well as psychopathy. Furthermore, the seven-repeat allele variant was highly present in a cohort of Russian and Chechen felons convicted for severe violent crimes, while a functional variant of the dopamine transporter was more associated with habitual offenders [141]. The same dopamine transporter gene variant was also found to be more prevalent in Pakistani inmates convicted of murder [142].

The degradation process of serotonin and dopamine occurs mainly through two enzymes—monoamino-oxydase (MAOA) and catechol-O-Methyltransferase (COMT) [143]. Both enzymes are known to have functional genetic variants linked to aggression and antisocial behavior, which have been studied extensively over the past decades [144]. Several upstream variable-number tandem repeat variations (uVNTRs) have been described in the promotor region of the MAOA gene, which controls its enzymatic activity. Of those, the two-repeat variant (2R; MAOA-low) is associated with lower transcriptional efficiency than the more common three- or four-repeat variants (3R or 4R; MAOA-high), resulting in different metabolic efficacies [143]. While MAOA occurs in both the degradation of serotonin and dopamine, COMT regulates exclusively extracellular dopamine levels by metabolizing dopamine into 3-Methoxytyramine (3-MT), which is in turn converted into homovanillic acid (HVA) by MAOA. The most common functional COMT polymorphism is Val108/158Met, where the amino acid methionine is replaced by valine with the Val/Val variant causing the enzyme to be two to four times more active [145].

In their landmark study from 2002, Caspi and colleagues [17] were the first to demonstrate an interaction between violence, adverse childhood events, and the MAOA-low variant. However, Ficks and Waldman [146] called the overall effect size of MAOA uVNTRs on antisocial behavior, including aggression, “modest” in their meta-analysis of 31 follow-up studies. Hence, it seems intriguing that there are criminal proceedings allowing MAOA-low genotyping as evidence, even resulting in lesser charges or sentences [147]. However, the most substantial evidence for the role of MAOA in aggression was demonstrated by MAOA knockout mice being more aggressive [148] and an observation made by Labonté and colleagues [149]. In the latter study, genetic modification of the expression of a long noncoding RNA regulating MAOA in the hippocampus led to a decrease in MAOA and an exacerbation of impulsive aggression in mice. Similar results were reported via pharmacological inhibition of MAOA during developmental phases in rodents [150]. Since pharmacological blockade of serotonin reuptake reduced the augmented aggression in MAOA-deficient mice, aggression may very well be a presynaptic serotoninergic effect rather than dopamine-mediated [151]. On the other hand, a recent study on 150 forensic psychiatric outpatients receiving aggression treatment cooperated the trauma and aggression interaction findings, but only for those patients with multiple traumas. Additionally, no effect on the treatment outcome was reported [152]. Nonetheless, a study a novel EEG based investigation pointed to hyper-responsiveness to threatening voices in MAO-low carriers [153] and increased aggressive reactivity to provocation [154] as well as morphological changes in the amygdala [155], while other studies in different ethnicities fully replicated Caspi’s findings [156,157]. To complicate matters more, gender may play a role as well. The MAOA × childhood experiences × aggression interaction had opposing effects in male and female participants and could be linked to amygdala activity during a reactive aggression task [158]. Finally, a study on male Italian prisoners reported dissuading results. Higher scores of aggression self-reports were observed in MAOA-low carriers, but only if they were exposed to physical neglect during growing-up; otherwise, the highest scores were those of MAOA-high carriers [159]. Similarly, Zhang et al. [160] reported the MAOA-high carrier to be the most aggressive in Chinese male adolescents, provided that a second polymorphism in the serotonin transporter gene was present.

The Valine/Methionine (Val/Met) COMT SNP research in human beings produced contradicting results regarding which variant (Val/Val or Met/Met) may influence more aggression or be linked to psychopathy [145,161], though the Met/Met-induced aggressive behavior was shown to be phenotypically preserved across species [162]. On the contrary, Hygen and colleagues [163] suggested that the Val/Val variant makes adolescents more malleable, rather than vulnerable, to adverse childhood experiences, leading to more aggressive behaviors. In line with this, male adolescent carrier for the Val/Val variant and with attention deficit disorder displayed poorer impulse control and reduced fear empathy [164]. Then again, Val/Val carriers in a Swedish population were associated with lower levels of physical aggression when exposed to violence compared to Met/Met carriers while still having a positive parent-child relationship [165]. Finally, the work of Fritz and colleagues [166] suggested a possible novel angle that may explain some of the dichotomous findings. In their study, a double SNP analysis focusing on the degradation pathway of dopamine was conducted in forensic inpatients. The adverse childhood x aggression interaction occurred only in participants carrying both the Val/Val highly active and the MAOA-low variants, suggesting that 3-MT may be responsible for the aggressive phenotype.

### 3.2. Glutamate

Glutamate is the most important excitatory neurotransmitter in the CNS and mediates its effect through two classes of receptors—ionotropic and metabotropic receptors. The most prominent ionotropic receptors are the α-Amino-3-hydroxy-5-methyl-4-isoxazolepropionic acid (AMPA) and Glutamate N-methyl-D-aspartate (NMDA) receptors. In the metabotropic receptor class, there are currently eight receptors in three functional groups described—mGluR_1_ and mGluR_5_ (group I), mGluR_2_ and mGluR_3_ (group II), and mGluR_4_, mGluR_6_, mGluR_7_, and mGluR_8_ (group III) [167].

In 2004, work from Vekovischeva and colleagues [168] first implicated a direct role of AMPA receptors in aggression, as mice with genetically deleted or functionally reduced AMPA receptors show significantly less inter-male aggressive behavior. Ten years later, a pharmacological approach with a systemic administration of an AMPA receptor antagonist corroborated those findings [169]. Interestingly, higher AMPA receptor levels were reported in the amygdala and the PFC in highly aggressive mice [170]. Finally, Zha et al. [171] demonstrated that projections from the VMHvl to the posterior part of the amygdala mediated aggression in an AMPA receptor-dependent manner.

Contrarily, targeting NMDA receptors to alter aggressive behavior appears to yield a somewhat biphasic response. Systemic ketamine administration, an NMDA receptor antagonist, decreased aggression at high doses but augmented it at low doses in adolescent mice [172]. Pharmacological blocking of NMDA receptors restricted to the PFC, however, had a diminishing effect of aggression in MAOA deficient mice solidly [173,174]. Targeting NMDA receptor subunits like GluN1 further strengthened these pharmacological findings, as GluN1 knockout mice displayed reduced aggressive behavior [175]. Furthermore, decreases in GluN2b subunit expression in the lateral amygdala was associated with aggression in socially isolated rodents [176].

Amongst the three groups of metabotropic receptors, interventions targeting group I had the most substantial effect. Blocking mGluR_5_ pharmacologically induced significant alterations in aggressiveness in both mice and hamsters [177,178]. Finally, recent work in mice implicated a small subpopulation of VTA glutamatergic neurons receiving strong inputs from the habenula to turn escape behavior into reactive, defensive aggression [179].

Somewhat surprising were the reported findings from Craig et al. [180]. In their study, ACC glutamatergic concentrations in the ACC were distinguishable according to the prevalent trait in the participants. Whereas callous-unemotional individuals had increased glutamate levels in the ACC, those showing proactive aggression had decreased levels. In line with these findings, another study investigating aggressive behavior in individuals with antisocial personality disorder found a positive correlation between glutamine (a precursor for glutamate) levels in the dlPFC and aggression [181]. However, Ende and colleagues [182] did not observe augmented ACC glutamate levels in aggressive test subjects with borderline personality disorder, though a positive correlation was shown with measures of impulsivity.

### 3.3. γ-Aminobutyric Acid (GABA)

GABA is the inhibitory neurotransmitter in the central nervous system and binds to two classes of receptors, the GABA_A_ receptor, a ligand-gated ion channel, and the GABA_B_-receptor, a metabotropic G protein-coupled receptor [183,184]. Both received substantial scientific attention in the context of aggression, especially since it became apparent that alcohol mediates some of its CNS effects through GABAergic receptors (for a summary of earlier research, please see [185]).

In mice, a recent systematic analysis combining microarray, quantitative polymerase-chain-reaction (PCR), and magnetic resonance imaging (MRI) based spectroscopy linked a 40% reduction of GABA in the ACC together with a 20-fold increase of the GABA-degrading enzyme *Abat* to excessive aggression in mice [186]. On the other hand, a pharmacology-based approach micro-infusing the GABA_A_ receptor agonist muscimol into the ACC demonstrated an increase in hyper-aggressiveness in mice [187]. Similarly, muscimol infusion into the LS changed the behavior of defeated Syrian hamsters from submissive to aggressive [188], an effect that was corroborated by Borland and colleagues [189] independent of the social experience of defeat. In line with these findings, muscimol- driven GABA_A_ receptor activation of the OFC yielded the same behavioral outcome in rats, suggesting that GABA_A_ receptors may play an important role in the modulation of aggression [41]. In a global genetic modification approach using point-mutated mice, Newman and colleagues [190] further specified the GABA_A_ receptors containing an α2 subunit to be mediating aggression. Aside from some methodological oddities, novel optogenetic stimulation of neuronal pathways connecting through or originating in GABAergic neurons in the medial amygdala pointed collectively towards a central GABAergic role in promoting aggression [18,80,191,192].

On a human level, the aforementioned preclinical findings appear to be translatable. In both patients with attention deficit disorder and borderline personality disorder, GABA levels in the ACC negatively correlated with self-rating aggression scores in an MRI-based spectroscopy approach [182]. Blood GABA concentrations in violent offenders with antisocial personality disorder positively correlated with aggression [193]. Furthermore, GABA_A_ receptor 2 gene polymorphisms rs279826 and rs279858 A-allele carriers were demonstrated to have an adverse stressful life event and aggression interaction [194].

### 3.4. Inflammatory Markers

Cytokines and prostaglandins have been demonstrated to act both as mediators of inflammation and neurotransmitters [195,196]. Hence, it is not surprising that several studies on cats were able to demonstrate a link between cytokine administration and augmented aggression [197,198,199]. In line with this, genetic deletion of the two tumor-necrosis-factor alpha receptors caused the durations of aggression to decrease [200]. Interestingly, a study based on a resident-intruder-challenge revealed that pharmacological or genetic alterations of interleukin-1 receptors in the dorsal raphe nucleus consistently increased aggressive behavior in mice [201]. Finally, lipopolysaccharide challenges in rats selected for the trait “aggressiveness” caused an augmented upregulation of interleukin-1 in the frontal cortex but a decrease in the hippocampus compared to controls [202].

In humans, studies from Coccaro and colleagues [203,204] were able to link peripheral interleukin-6 to impulsive-aggressive subjects and increased cerebrospinal fluid levels of the soluble interleukin-1 receptor II to aggressive patients with personality disorders. Earlier work of Provencal and others [205] found the anti-inflammatory cytokines interleukin-4 and interleukin-10 to be lower in children with aggression issues. However, a recent study failed to find an association between functional SNPs within interleukin-1beta, interleukin-2, and interleukin-6 in aggressive children [206].

### 3.5. Opioid Receptors

The mu(µ)-opioid receptor 1 (OPRM1) is commonly regarded as the most relevant opioid receptor for aggression. Especially its functional single nucleotide polymorphism C77G (corresponding to A118G in human beings) was linked to augmented cortisol responses and heightened aggressive behavior in rhesus monkeys [207]. Driscoll and colleagues [208] further supported this interaction, linked it to serotonin, and expanded it as a predictor of alcohol-stimulated aggression. It is also worthwhile mentioning that heroin addicted rats produced more aggressive offspring than their control group, which likely suggests that µ-opioid receptor activation causes aggressive behaviors [209]. In another study, Weidler et al. [210] demonstrated a correlation between the OPRM1 SNP A118G and physical aggression in human beings. A very recent work of Cimino and colleagues [211] was also able to associate the A118G SNP with disruptive behavior and limitation in mood regulation in children.

### 3.6. Orexin

Preclinical and clinical research also indicates a central role for orexin in the control of aggressive behavior. Recent work from Flanigan and colleagues [212] implicated orexin signaling via the orexin 2 receptor (OxR2) on GABAergic cells in the lateral habenula to play a role in intermale aggression in mice. Previously, the same group has also suggested orexin to control the valence of aggression through a strong habenula-to-VTA neuronal connectivity in mice [213].

In humans, the HCRTR1 rs2271933 A/A carrier genotype was associated with heightened aggressive behavior [214]. Such genotype-phenotype interaction was argued to be based on an augmented reward association with violence [215].

### 3.7. Oxytocin/Vasopressin

Oxytocin and vasopressin are fundamentally linked and guide the regulation of social behavior, such as parental nurturing and social cognition, but they also relate to affective states and emotional discrimination [216,217,218]. Over the last decades, the hormone vasopressin has received a great amount of attention in the context of aggression (for a detailed review, see [219]). For instance, vasopressin release patterns were demonstrated to differ in low and high-anxiety rat strains across brain areas in the context of intermale aggression [220]. Furthermore, glutamate-vasopressin interaction in the lateral hypothalamus augmented steroid-induced aggression in Syrian hamsters [221]. Conversely, genetic deletion of the vasopressin receptor 1b (V1b) reduced aggression in male mice [222]. Somewhat counterintuitive, heightened oxytocin levels in the paraventricular nucleus (PVN) and the amygdala was also linked to high levels of maternal aggression in lactating rats [223]. The interplay between oxytocin and vasopressin continued to be complex, as demonstrated in recent work in the ventral and dorsal LS [224]. Using a mixed neuropharmacological, optogenetic, and chemogenetic approach, Oliveira and colleagues showed that an increase in oxytocin level in the ventral LS and a decrease in vasopressin level in the dorsal LS were associated with aggressive behavior in rats. Strikingly, systemic administration of both vasopressin and oxytocin suppressed isolation-enhanced aggressive behavior in male mice in the social interaction test [225].

On a human level, intranasal administration of vasopressin caused an increase in “preemptive strikes” in a computer simulation in both male and female participants [226]. In test subjects with high Machiavellian traits, a combined administration of testosterone and vasopressin augmented punishing behavior during a provocation paradigm [227]. In a different study, even though increased activity in the right superior temporal sulcus was measured during a competitive reaction game, intranasal vasopressin administration failed to alter the behavior in the test group [228].

## 4. Alcohol and Aggression: Insight from Preclinical Studies

The fact that aggression between members of the same species increases with alcohol consumption has been repeatedly demonstrated [229]. Emerging trends from preclinical studies have shown that alcohol induces aggressive behavior by reducing the ability to avoid dangerous stimuli, especially due to its inhibitory effect on anxiety-related behaviors, leaving an organism more exposed to harmful attacks [229]. However, reports investigating the effect of alcohol on aggressive behaviors have not always been consistent. Rats trained to drink a 10% solution of alcohol and housed in a visible burrow system showed decreased levels of aggression before and during housing, most likely reflecting a change in their social behaviors [230]. At high doses, alcohol was also shown to decrease aggressive behavior in laboratory animals, reflecting a sedative action and inhibitory action on muscular activity [229]. Aggressive behaviors of laboratory animals are, however, greatly increased when exposed to low or moderate doses of alcohol [231,232,233]. Various studies have shown the dose of 1.0 g/kg alcohol (6% *w/v*) to be effective at producing alcohol-induced aggression [234,235,236].

### 4.1. Alcohol and GABA

Interestingly, alcohol’s effect on aggression can be influenced by several neuromodulators, among them the inhibitory neurotransmitter gamma-aminobutyric acid (GABA). Indeed, there is a wide range of evidence showing that changes in GABA receptor function may play a role in the behavioral effect of alcohol [237,238]. In a study using male CFW mice, Fish and colleagues [235] showed that alcohol or the endogenous positive modulator of GABAa receptor, allopregnanolone, increases aggression when administered alone, but when administrated together and at certain doses, one suppresses the effect of the other. Similarly, combined administration of a low dose of alcohol and chlordiazepoxide, which also acts as a positive modulator of GABAa receptor, led to significant increases in alcohol-heightened aggression in male mice to a larger extent than when either drug was administered alone [239]. Conversely, alcohol-heightened aggression is reduced by the administration of GABAa receptor antagonists in rodents and non-human primates [232]. Such interaction between alcohol and various modulators for GABAa receptors likely points to a key role of the GABAergic system in the mechanisms underlying alcohol-heightened aggression and is in line with findings from electrophysiological recordings showing that the stimulation effect of alcohol is linked to changes in GABA-receptor mediated responses [240].

### 4.2. Alcohol and Serotonin

Another mechanism by which alcohol induces aggression is through the involvement of the serotonin system. In experimental mice, activation of 5-HT1A [241] or 5-HT1B [242] receptors with a selective agonist decreased the aggression-heightened effect of alcohol in mice. Although findings from these studies failed to clearly determine whether effects were due to the action at somatodendritic 5-HT autoreceptors or heteroreceptors, they provided evidence for the serotonergic system as a critical site in the regulation of alcohol-heightened aggression. Findings from preclinical studies have also shown that reduced serotonin function is linked to impaired impulse control [243] and decreased anxiety [244], raising the possibility that the alcohol-heightened effect is likely linked with a deficiency in brain serotonin levels. Conversely, previous studies have shown that brain 5-HT levels in experimental animals are decreased following withdrawal from alcohol, and increased following chronic alcohol intake [245], underlying a high degree of complexity of the mechanisms by which the serotonergic system contributes to the aggression-heightened aggression. More recently, it was also shown that the administration of an inhibitor of tryptophan hydroxylase (which, as previously mentioned, inhibits serotonin synthesis) or an antagonist to 5-HT1A, 5-HT1B, or 5-HT2A receptors led to a marked reduction in alcohol-induced aggressive episodes in zebrafish [246]. The serotonergic system has also been implicated in the neural circuit of fear and anxiety [247,248]. Reduced anxiety has typically been observed upon exposure to certain doses of alcohol [246,249,250] and is correlated with increased aggression in experimental animals [246,251]. In an animal model of Zebrafish, administration of a 5-HT1B agonist was shown to mimic the anxiolytic-like response induced by alcohol, an effect that was abolished in the presence of an inhibitor of tryptophan hydroxylase or an antagonist to 5-HT1B receptors [246]. In addition, infusion of a 5-HT1B receptor agonist into the medial PFC of male mice after alcohol drinking was associated with increased aggressive behavior [234]. These findings point to a key role of the 5-HT1B receptor in the anxiolytic effect of alcohol, though the involvement of other serotonergic receptors remains to be fully explored.

### 4.3. Alcohol, Dopamine, and Behavioral Sensitization

Alcohol-heightened aggression was also linked to changes in the dopamine neurotransmitter system. Alcohol activates dopaminergic circuits in several regions of the brain, including the nucleus accumbens [252], frontal cortex [253], and amygdala [254], and this effect is thought to play a role in its action over aggressive behavior. Rats that freely self-administer alcohol show an increase in dopamine accumbal release, and this effect persists during a subsequent aggressive confrontation with an intruder [255,256]. Alcohol consumption on a regular basis can also lead to a phenomenon known as behavioral sensitization and may impact how the drug affects aggressive behavior [233,257]. In fact, repeated administration of alcohol may increase the motor-stimulating effects of this drug and can make mice more susceptible to increased aggressiveness following alcohol abuse [233]. Furthermore, alcohol-induced aggression is related to some of the neuroadaptations brought on by repeated alcohol consumption [233]. Sensitization to drugs of abuse has often been linked to changes in dopamine neurotransmission. Most drugs of abuse, such as psychostimulants [258,259], opiates [260], and nicotine [261,262], have been associated with sensitization of the mesolimbic dopamine circuit. Similarly, several findings have demonstrated the involvement of mesolimbic dopamine signaling in alcohol-induced sensitization [263,264]. However, although behavioral sensitization to alcohol has been extensively examined in preclinical studies [265], only a few studies have examined its relevance to aggressive behaviors and its potential translation to human models.

### 4.4. Alcohol and Glutamate

Alcohol may also act on the glutamatergic system within certain brain circuits to promote aggression in laboratory animals. In one study conducted by Newman and colleagues [266], mice were trained to self-administer 1.0 g/kg of alcohol and were characterized as either alcohol-heightened or alcohol-non-heightened aggressors during resident-intruder confrontations. In mice classified as alcohol-heightened aggressors, the GluN2D subunit of the NMDA receptor was overexpressed in the prefrontal cortex (PFC), suggesting that alcohol may act on the cortical circuit to promote aggression [266]. Interestingly, the authors of the study also showed that intraperitoneal administration of the NMDA receptor antagonists, ketamine or memantine, increased aggression in alcohol-heightened mice when prevented from accessing alcohol and that this effect was blocked by prior alcohol intake [266]. On the other hand, injection of memantine in the prelimbic medial PFC interacted with prior alcohol intake to aggravate aggressive behavior in the non-heightened aggressor mice [266]. In line with these findings, prior preclinical studies showed that memantine could facilitate aggressive behavior in mice after self-administration of alcohol [267] or during alcohol withdrawal [268]. The NMDA receptor antagonist, ketamine, was also shown to increase aggressive behavior in rats when administered intraperitoneally [269]. In addition, the extracellular level of glutamate in the medial PFC was significantly increased after 8 weeks of alcohol withdrawal in mice [268]. Together, these findings likely point to a role of NMDA receptors within the PFC in alcohol-related aggression and that these receptors could potentially act as targets for therapeutic interventions.

## 5. Alcohol and Aggression: Insight from Clinical Studies

**Figure 2:**
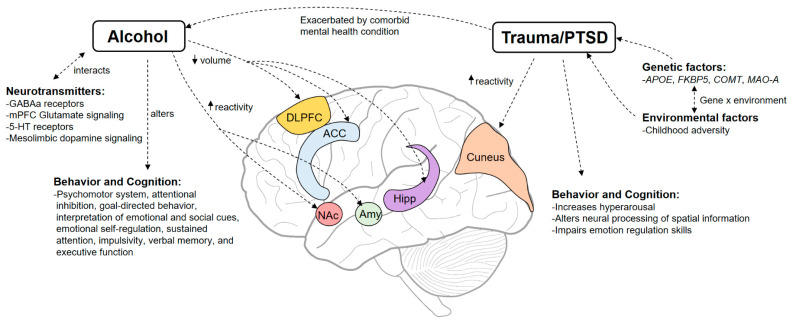
Mechanisms through which alcohol and trauma lead to aggressive behavior. Alcohol consumption could lead to increased aggression by interacting with neurotransmitter systems, causing structural or functional changes in certain brain regions, or altering certain aspects of behavior and cognition. Alcohol consumption can be precipitated by trauma exposure and is exacerbated in individuals with comorbid mental health conditions. Genetic factors can interact with environmental factors, such as childhood adversity to increase the likelihood of developing PTSD. Trauma exposure, in turn, can lead to functional changes in brain regions and lead drastic changes in behavior and cognitive functions. Abbreviations: Anterior Cingulate Cortex (ACC); Amygdala (Amy); Dorsolateral prefrontal cortex (dlPFC); Hippocampus (Hipp); Medial Prefrontal Cortex (mPFC); Nucleus Accumbens (NAc).

Alcohol use could pose a serious threat to society, with evidence showing higher rates of alcohol consumption to be associated with higher rates of homicide and violent crimes [270,271]. Alcohol use can lead to harmful effects both to the drinker and others; for instance, a study assessing violence-related injuries across 14 countries showed that at least 60% of the injuries involved alcohol use on the part of the victim, the perpetrator, or both [272]. Several other studies have demonstrated a strong positive correlation between alcohol abuse and violence [273,274,275], especially domestic violence, where married alcoholic men were at least six times more likely to commit domestic violence compared to others [276]. Dose-dependent studies of alcohol-related aggression in humans (using doses from 0 to 1.0 g/kg) also revealed a highly significant positive linear relationship between alcohol dose and aggression in both men and women [277,278]. The effects of alcohol on aggressive behaviors in humans are thought to be caused by a plethora of factors, including alterations in the psychomotor system, modifications of the stress and anxiety level, changes in cognitive and emotional processing, as well as disruption of neurotransmitter systems, all of which are discussed below and illustrated in Figure 2 (Also for review see [279]).

### 5.1. Behavioral Effects and Risk Factors of Alcohol

Several studies have shown an association between alcohol use and the disruption of the psychomotor system with some degree of variation depending on the complexity of the task being analyzed [280,281]. Addictive substances such as alcohol may disrupt the psychomotor system and heighten motor activities such as approach, sensation seeking, and attacks, thus increasing one’s potential aggressive behavior [282]. Same as other addictive drugs [283], alcohol may have disinhibiting effects on behavior as it induces a premature response based on preliminary stimulus evaluation in the “go/no go” verbal recognition task, reflecting its impulsive and psychomotor stimulant nature [284]. Such deficits in behavioral response inhibition have also previously been correlated with aggressive behaviors [285,286]. Alcohol was also shown to impair attentional inhibition, that is, the ability to ignore distracting environmental stimuli in order to focus on relevant information [287]. Despite its numerous effects on behavior, alcohol may not encourage aggressive behaviors equally in all situations. For example, alcohol-induced aggression is regulated by situational factors like provocation [288], social factors [289], and drinking environment [290]. The propensity toward alcohol-induced aggression may be individually driven by personal expectations of the effects of alcohol, by previous experiences of violent behavior, and by the perpetrator’s traits and attitude [289,291]. A better understanding of risk factors for alcohol-induced aggression may help better understand its effect on behavior and could pave the way for the development of new individualized therapies.

### 5.2. Effect of Alcohol on Sensory, Emotional, and Cognitive Processes

There is mounting evidence that alcohol consumption can escalate aggressive behavior by impairing sensory, emotional, and cognitive functioning [289,292,293]. It has been proposed that alcohol could reduce stress levels owing to its anxiolytic effect, hence ’hampering one′s ability to avoid threatening situations and increasing his tendency to engage in hostile behaviors [293]. Alcohol can also alter how individuals interpret emotional and social cues, making them more likely to misinterpret danger and provocation, and may also lead to aggressive behavior by influencing emotional self-regulation (For review, see [293,294]). In addition, evidence suggests that alcohol may increase the likelihood of aggressive behaviors by altering cognitive functions. In a study employing healthy social workers, Marinkovic and colleagues [295] showed that individuals with alcohol consumption (using a dose of 0.6 g/kg for men and 0.55 g/kg for women) failed to maintain complete self-control because of altered goal-directed behavior. In another study employing university students, Magrys and Olmsted [296] found that medium (0.6 g/L) and high (0.9 g/L) blood alcohol concentrations (BAC) impaired sustained attention, impulsivity, and verbal memory, with medium doses having greater damage, whereas low doses (0.2 g/L BAC) had no effect. These findings illustrate an inverted U-shaped correlation between alcohol doses and cognitive deficits [296]. Alcohol-related aggression was also proposed to be mediated by executive functioning in as much as alcohol intoxication impairs these cognitive processes and is more likely to facilitate aggressive behaviors in individuals with low, rather than high, executive function [297].

### 5.3. Alcohol, Neurotransmitter Systems, and Brain Regions

There is numerous evidence showing that the serotonergic neurotransmission is compromised by prolonged alcohol use, which affects how the limbic system processes aversive stimuli and how the prefrontal cortex controls behavior [292,293]. Studies employing functional magnetic resonance imaging (fMRI) and manipulating central serotonin levels using acute tryptophan depletion show that individuals with low serotonin levels have altered processing of emotional facial expressions [298,299]. Impairments in facial emotional perception have been associated with aggressive and anti-social behavior [300,301] and may explain the increased tendency of hostile behavior after alcohol consumption. Consistently, impaired processing of emotional faces has been associated with long-term alcoholism and a deficiency in the activation of the amygdala and hippocampus [302], known to be involved in stress and memory formation, respectively [303,304]. In another study, low serotonin activity has been linked to increased suicide tendency in depressed individuals with comorbid alcoholism; however, no differences in aggressive–impulsive traits were observed between low and high-lethality suicide attempters [305]. Alcohol-related aggression is also significantly affected by the interaction of serotonin neurotransmission with the GABAergic system and by genetic and environmental factors [292]. Additionally, the GABAa receptor α2 subunit single nucleotide polymorphism rs9291283 has been linked to alcohol-dependent aggression in participants of Croatian origin [306].

Last but not least, there are numerous findings from neuroimaging studies showing that alcohol consumption leads to structural and functional changes in certain brain regions (For review, see [307]); however, studies directly assessing the neural correlate of alcohol-related aggression are very limited. In one study using MRI in alcohol-dependent individuals, alcohol was shown to cause significant regional volume reductions in the anterior cingulate cortex (ACC), dorsolateral prefrontal cortex (DLPFC), and hippocampus, whereas short- or long-term abstinence caused a significant volume increase in the ACC, DLPFC, orbitofrontal cortex (OFC), and hippocampus [308]. The study did not directly assess the relationship with aggression; however, since many of these regions are involved in executive control and emotional processing, the increased aggressive behavior observed in alcoholics is likely to be attributed, at least partly, to changes in the activation of such regions. In another study using young, healthy, sober adults, alcohol administration (using a moderate dose of 0.6 g/kg) led to marked increases in reactive (provoked) aggression and correlated to increased reactivity of the amygdala and ventral striatum [309]. These findings suggest the involvement of the amygdala and the ventral striatum in the stressful response elicited by provoked aggression and potentially suggest the involvement of dopaminergic circuits, which heavily innervate the ventral striatum [310,311] in human alcohol-induced reactive aggression. However, more work is needed to better address the neural correlation of alcohol-related aggression in humans.

## 6. Role of Trauma in Alcohol Consumption and Aggressive Behavior

Traumatic life experiences, such as physical and sexual abuse and the death of a loved one, occur at an alarming rate worldwide, and are considered a major public health issue with detrimental effects on both physical and mental health [312,313]. Although some individuals with prior trauma exposure can become quite resilient [314], early trauma exposure is well known to increase the risk for a number of psychiatric disorders, such as depression, anxiety, and posttraumatic stress disorder (PTSD) [315]. PTSD can occur at a rate of approximately 5–10%, depending on the type of traumatic experience and the gender of the individual [316]. In a series of epidemiological surveys administered to 24 different countries, traumatic experiences such as sexual assaults or being stalked have been associated with a higher risk of developing PTSD than the unexpected death of a loved one [317]. In addition, in a meta-analysis evaluating the rates of PTSD in trauma-exposed children and adolescents, boys exposed to non-interpersonal trauma had a reduced risk of developing PTSD compared to girls exposed to interpersonal trauma [318]. According to the Diagnostic and Statistical Manual of Mental Disorders, Fifth Edition (DSM-5), which is the standard classification of mental disorders, the criteria for PTSD include exposure to a traumatic event such as actual or threatened death, serious injury, or sexual violence, followed by symptoms grouped in four categories: intrusion, avoidance, negative thoughts and mood, and changes in arousal and reactivity [319]. The first category of symptoms, intrusion, refers to the reappearance of the traumatic event, such as in flashbacks and/or dreams, and the increased physical reactivity when reminded of the event. The second category, avoidance, relates to all behavior that aims at avoiding individuals, places, or situations that are related to the traumatic event. The third category of symptoms, negative thoughts, and mood, involves the feeling of shame, fear, guilt and anger, whereas the last category involves difficulty sleeping, hypervigilance, increased irritability, and impulsive and self-destructive behavior [319]. Being exposed to a traumatic event might affect the individuals at a psychological, physical, emotional, cognitive or behavioral level. Individuals with PTSD also often exhibit comorbid mental health conditions and may rely on drugs of abuse to cope with their symptoms [320], further exacerbating their behavior and cognitive functions. A better understanding of the symptomatology of PTSD could help clinicians, and other health professionals adopt the most effective therapeutic practice and healing strategy. The next sections discuss findings highlighting the interrelationship between trauma, alcohol use, and aggression and provide the current state of knowledge regarding the effect of trauma on behavior and cognition as well as the genetic and environmental factors that could lead to PTSD (also see Figure 2).

### 6.1. Trauma and Alcohol Consumption

Traumatic experiences have also been linked to substance use disorders, including alcohol abuse; individuals who suffered from childhood trauma have high rates (up to 60%) of alcohol misuse [320,321] compared to the general population (up to 10%) [322,323]. Research has consistently shown that individuals with posttraumatic stress symptoms are more likely to consume alcohol as a coping strategy to manage distress and anxiety (See [320]). In particular, the prevalence of comorbid alcohol misuse in individuals diagnosed with PTSD ranged from approximately 10–61%, whereas the prevalence of comorbid PTSD in individuals with alcohol misuse ranged from approximately 2–63% [320]. In a study employing 294 adult women substance abusers, Stewart and colleagues [324] showed that PTSD symptoms were positively correlated with the frequency of alcohol drinking in negative contexts (i.e., associated with unpleasant emotions) but not in positive contexts (i.e., associated with pleasant emotions). Similarly, in another study examining predictors of alcohol misuse in Iraq and Afghanistan war veterans, individuals with a positive diagnosis of PTSD were two times more likely to report alcohol misuse, especially individuals having emotional numbing symptoms [325].

Several factors may contribute to the link between PTSD and increased alcohol consumption. One of these is that individuals with PTSD often have high levels of distress, anxiety, and depression [326,327]. These symptoms can be overwhelming and may impact an individual’s ability to function in daily life. Alcohol use can therefore serve as a way to self-medicate or cope with these symptoms [328]. Individuals with PTSD may have impaired emotion regulation skills, making it more challenging to manage negative emotions [329]. However, while alcohol use may provide temporary relief, it can also worsen the severity of PTSD symptoms over time, leading to a cycle of dependence. Research has shown that veterans with PTSD and alcohol use disorder are more likely to experience signs of depression, anxiety, and suicidal ideation than those with alcohol use disorder only [330]. Likewise, veterans with comorbid PTSD and alcohol use disorder are more than three times as likely to attempt suicide compared to veterans with PTSD only [330]. In addition, alcohol use can interfere with the effectiveness of PTSD treatments, making it more difficult for individuals to manage their symptoms and recover from the traumatic event [331]. Treatment for individuals with comorbid PTSD and alcohol use disorder is complex and requires a comprehensive approach. Several evidence-based treatments, including cognitive-behavioral therapy and prolonged exposure therapy, have shown efficacy in treating PTSD and reducing alcohol consumption [332]. Pharmacological interventions, such as naltrexone and acamprosate, have also been shown to be effective in reducing alcohol consumption in individuals with comorbid PTSD and alcohol use disorder (as reviewed in [333]).

### 6.2. Trauma and Aggressive Behavior

Several studies have shown a significant association between trauma, PTSD, and aggressive behavior [334]. For example, there are reports indicating that individuals with a history of trauma and PTSD are significantly more likely to engage in intimate partner violence than those without trauma or PTSD [335,336]. In another study employing first-episode psychosis with a history of trauma, not only did researchers find that trauma was associated with depression severity and suicide ideation, but they also showed that it was linked with aggression, aggression severity, and the type of aggression [337]. Specific symptoms of PTSD, such as hyperarousal, may also be particularly associated with aggressive behavior. For instance, Elbogen and Johnson [338] found that hyperarousal symptoms of PTSD were significantly associated with violence perpetration among Iraq and Afghanistan war veterans. Similarly, Bomyea and colleagues [339] found that hyperarousal symptoms were positively associated with aggression in individuals with PTSD. Early trauma and secondary psychopathy were also shown to serve as significant predictors of impulsive aggressive behaviors in male veterans [340]. In another study employing veterans with deployment experience, anger and aggression problems were associated with increased activation in the cuneus, a brain region associated with visuospatial processing, suggesting that traumatic experiences may lead to aggressive behavior by altering neural processing of spatial information [88]. Such findings add to the growing body of the research on the neural underpinnings of aggression and have implications for the development of targeted interventions for individuals with anger and aggression problems.

The relationship between trauma, PTSD, and aggressive behavior is complex and can be influenced by a range of individual and contextual factors. For example, comorbid mental health conditions, such as substance use disorders or mood disorders, may exacerbate the link between trauma/PTSD and aggressive behavior [341,342]. Childhood emotional and physical neglect were also found to be significant correlates in the development of posttraumatic aggression and self-harm [343]. In terms of treatment, several interventions have proved effective in reducing aggressive behavior among individuals with trauma or PTSD. Cognitive-behavioral therapy and prolonged exposure therapy are two evidence-based therapies that have been shown to be effective in reducing PTSD symptoms and aggressive behavior [344], which may potentially alleviate symptoms reminiscent of PTSD including aggressive behavior. Pharmacotherapies, such as selective serotonin reuptake inhibitors (SSRIs) [345], which are used for the first line of treatment of depression, or antipsychotics [346], may also be used to effectively reduce aggression in individuals with PTSD.

### 6.3. Genetic Factors in Trauma, Alcohol Consumption, and Aggression

Genetic factors have been shown to play a role in the development of trauma, alcohol consumption, and aggressive behavior. While PTSD, aggression, and alcohol use can have complex environmental and psychological causes, studies have shown that individual differences in genetics can influence susceptibility to these issues. For instance, twin studies have shown that genetic risk factors could account for approximately 30–40% of the heritability of PTSD [347] and 50% for alcohol use disorder [348] and aggression [349]. Furthermore, several studies have examined the role of genetic factors in the relationship between trauma exposure, alcohol consumption, and aggression. In a study employing Korean veterans and investigating polymorphisms in the apolipoprotein E gene (APOE), higher frequencies of APOE ε2 alleles were found in the PTSD group, suggesting that this genetic variation operates as a susceptibility factor for PTSD [350]. In addition, a significant interaction effect was found between harmful drinking and the absence of the ε2 allele associated with PTSD risk, suggesting that the APOE gene regulates alcohol use and its interaction with aggressive behavior in PTSD [350]. There is also evidence of gene × environment effect on the risk of developing PTSD. For instance, genetic variations in the *FKBP5* gene, which is involved in regulating the body’s response to stress, can interact with childhood adversity to increase the risk of developing PTSD symptoms [351,352]. Other genetic polymorphisms, such as those in the catecholamines converting enzymes COMT and MAO-A, can interact with traumatic childhood experiences to increase aggression [166,353]. However, despite significant progress in research, future research is needed to better understand the role of genetics in these issues and to develop more effective preventive and treatment strategies.

## 7. Conclusions and Future Directions

The aggression literature is large and diverse, with an overall consensus indicating a positive relationship between alcohol consumption and traumatic experience. Findings from preclinical and clinical studies have provided useful insights into understanding the neurobiological and genetic basis involved in this linkage. Despite this, important limitations remain, including differences in the targeted population and failure to identify and control for factors moderating this relationship. Preclinical studies investigating the relationship between alcohol, trauma, and aggression have also largely been limited by the degree to which they could mimic human behavior. In particular, methods of forced alcohol exposure in preclinical models do not reliably mimic the human condition, and the amount of aggressive behavior observed may not always be consistent with the amount of alcohol ingested, making it difficult to interpret data and translate them into the clinic [231]. Problems with the transferability of animal findings to humans could significantly impact pharmacotherapy development and should be urgently considered in future studies by adopting the correct model organism and a disease model that conforms to the DSM-5 criteria. The aggression literature also suffers from problems of data replication due to publication bias for positive results and low power of effect size with small samples. Future studies should include larger sample sizes for more accurate and reproducible results. Furthermore, gender differences in aggressive behaviors are poorly understood and need further elucidation. Finally, to date, most of the studies concerned with examining the neurobiological effect of alcohol consumption or symptoms following a traumatic experience have largely assessed their effect on certain brain regions or neurotransmitter systems without making a correlation with aggressive behavior. More work is clearly needed to better assess the neurobiological correlation for the interaction between trauma, alcohol consumption and aggressive behavior. Future studies should also focus on better understanding the role of genetics, and its complex interaction with environmental factors, in the development of trauma, alcohol consumption, and aggressive behavior, as this could open avenues for early targeted intervention and preventive strategies.

## Data Availability

Not applicable.
